# Hsa_circ_0002062 Promotes the Proliferation of Pulmonary Artery Smooth Muscle Cells by Regulating the Hsa-miR-942-5p/CDK6 Signaling Pathway

**DOI:** 10.3389/fgene.2021.673229

**Published:** 2021-07-12

**Authors:** Yali Wang, Xiaoming Tan, Yunjiang Wu, Sipei Cao, Yueyan Lou, Liyan Zhang, Feng Hu

**Affiliations:** ^1^Department of Respiratory Medicine, Renji Hospital, School of Medicine, Shanghai Jiao Tong University, Shanghai, China; ^2^Department of Thoracic Surgery, The Affiliated Hospital of Yangzhou University, Yangzhou University, Yangzhou, China; ^3^Clinical Medical College, Yangzhou University, Yangzhou, China; ^4^Department of Cardiology, Renji Hospital, School of Medicine, Shanghai Jiao Tong University, Shanghai, China

**Keywords:** circular RNAs, microRNAs, pulmonary artery smooth muscle cells, CDK6, hypoxia-induced pulmonary hypertension

## Abstract

Currently, new strategies for the diagnosis and treatment of hypoxia-induced pulmonary hypertension (HPH) are urgently required. The unique features of circRNAs have unveiled a novel perspective for understanding the biological mechanisms underlying HPH and the possibility for innovative strategies for treatment of HPH. CircRNAs function as competing endogenous RNAs (CeRNA) to sequester miRNAs and regulate the expression of target genes. This study aimed to explore the roles of hsa_circ_0002062 on the biological behaviors of pulmonary artery smooth muscle cells (PASMCs) in hypoxic conditions. A number of *in vitro* assays, such as RNA-binding protein immunoprecipitation (RIP), RNA pull-down, and dual-luciferase assays were performed to evaluate the interrelationship between hsa_circ_0002062, hsa-miR-942-5P, and CDK6. The potential physiological functions of hsa_circ_0002062, hsa-miR-942-5P, and CDK6 in hypoxic PASMCs were investigated through expression modulation. Our experiments demonstrated that hsa_circ_0002062 functions as a ceRNA, acts as a sponge for hsa-miR-942-5P, and consequently activates CDK6, which further promotes pulmonary vascular remodeling. Therefore, we speculate that hsa_circ_0002062 could serve as a candidate diagnostic biomarker and potential therapeutic target for HPH.

## Introduction

Hypoxic pulmonary hypertension (HPH) presents as an elevated mean pulmonary artery pressure, results from hypoxic pulmonary vasoconstriction and abnormal vascular remodeling, and ultimately leads to right ventricular hypertrophy and heart failure (Rowan et al., 2016). HPH is characterized by pathological changes such as the proliferation of pulmonary artery smooth muscle cells (PASMCs), pulmonary artery endothelial cells, and fibroblasts (Kylhammar and Rådegran, 2017). Despite the application of recent advances in the treatment of HPH, patients with this disease experience a poor median survival rate (Li et al., 2019). Hitherto, many signaling cascades have been detected to play a role in HPH and hold great promise as potentially useful targets for therapeutic intervention (Gassmann et al., 2020). Nevertheless, treatment options remain limited, and in-depth elucidation of the detailed molecular mechanisms underlying vascular remodeling in HPH is urgently required (Smith et al., 2020). Moreover, the function of new non-coding RNAs in the progression of HPH remains largely unknown.

Circular RNAs (circRNAs) are a novel type of non-coding RNA with a covalently closed loop where the 3′ and 5′ ends are joined together (Di Timoteo et al., 2020). The function of circRNAs is just beginning to be unraveled. Growing evidence has provided strong support for the notion that circRNAs play a fundamental role in regulating various physiological and pathophysiological processes (Jahng et al., 2020; Wawrzyniak et al., 2020). However, their function and molecular mechanism in pulmonary hypertension remains an enigma and awaits further in-depth study. CircRNAs are regarded as competing endogenous RNAs that sequester microRNAs (miRNAs) by complementary base pairing (Ghafouri-Fard et al., 2020; Liu et al., 2020). miRNAs are featured as a class of small non-coding RNAs and suppress gene expression by promoting mRNA degradation or inhibiting mRNA translation, and thus, represent novel prospective targets for the diagnosis and therapeutics of various diseases (Barlak et al., 2020).

Previous evidence has demonstrated that mmu_circ_0000790 binds to miR-374c and acts as an endogenous miR-374c sponge by activating the Notch pathway via FOXC1 in mice model with HPH (Yang et al., 2020). A recent study illustrated the functionality of hsa_circ_0002062 as a key circRNA for patients with chronic thromboembolic pulmonary hypertension (CTEPH) through high-throughput sequencing and bioinformatics analysis (Miao et al., 2017). This study showed that hsa_circ_0002062 bound to hsa-miR-942-5P and acted as a miRNA sponge to inhibit hsa-miR-942-5P activity, which led to the increase of CDK6 expression, a target for hsa-miR-942-5P. CDK6 has been found to regulate the cell cycle and promote cell proliferation. Hence, downregulated hsa_circ_0002062 has been implicated as a prospective therapeutic target in PH. The hsa_circ_0002062/hsa-miR-942-5P/CDK6 signaling pathway may be involved in PH, but a detailed mechanism has not been elucidated yet. Based on the aforementioned studies, we hypothesized that the potential interactions between hsa_circ_0002062, hsa-miR-942-5P, and CDK6 might influence cell proliferation in the progression of HPH.

## Materials and Methods

### Cell Lines and Cell Culture

Human pulmonary artery smooth muscle cells (hPASMCs) were purchased from Lonzapet (Lonza, Switzerland). All of the cells were cultured in DMEM (HyClone Victoria, Australia) supplemented with 10% fetal bovine serum (Gibco, Carlsbad, CA, United States), and 1% penicillin/streptomycin at 37°C in incubators containing 5% CO_2_. PASMCs was randomly divided into a hypoxic group (3% O_2_, 5% CO_2_, and 92% N_2_) and a normoxic group (21% O_2_, 5% CO_2_, and 74% N_2_), which were maintained in hypobaric and normoxic incubators, respectively.

### Quantitative Real-Time PCR Analysis

According to the manufacturer’s protocol, total RNA was extracted from hPASMCs using the Trizol reagent (Invitrogen, Carlsbad, CA, United States). RNA samples were digested with Ribonuclease R (Geneseed, China) to degrade linear RNA and improve the purity of circRNA. The expression of hsa-miR-942-5P was measured using TaqMan miRNA assays based on the provided instructions, U6 was used for normalization. For the analysis of the levels of hsa_circ_0002062 and CDK6, glyceraldehyde 3-phosphate dehydrogenase (GAPDH) was used as an internal control. Reverse transcription (RT) was conducted using the Revert Aid^TM^ First Strand cDNA Synthesis Kit (Fermentas, United States). Amplification reactions were performed with an ABI PRISM 7300 Fluorescent Quantitative PCR System (Applied Biosystems, Foster City, CA, United States). The primers used in the study were synthesized by Beijing Genomics Institute (BGI) (Shenzhen, China). All procedures were performed in triplicate, and the sequences of the forward and reverse primers are as follows: Hsa_circ_0002062 Primer F 5′TTATTGACTGGGTCTTCC3′, Primer R 5′CATACATACATACATAGGGTG 3′; CDK6 Primer F 5′ TAACCTCAGTGGTCGTCAC 3′, Primer R 5′GTCTT TGCCTAGTTCATCG 3′;

GAPDH Primer F 5 GGATTGTCTGGCAGTAGCC 3′, Primer R 5′ATTGTGAAAGGCAGGGAG3′; hsa-miR-942-5p RT Primer, 5′GTCGTATCCAGTGCAGGGTCCGAGGTATTCG CACTGGATACGACCACATG 3′, PCR Primer, Primer F 5′CG CGTCTTCTCTGTTTTGGC 3′, Primer R 5′AGTGCAGGGT CCGAGGTATT 3′; U6 Primer F 5′CTCGCTTCGGCA GCACA 3′,Primer R 5′AACGCTTCACGAATTTGCGT 3′. The expression of target genes was measured using the relative quantitative method (2^–ΔΔCt^).

### RNA Interference and Plasmid Constructs

shRNAs against CDK6 as well as an hsa-miR-942-5P inhibitor, an hsa-miR-942-5P mimics, and a negative control shRNA, were purchased from Gene Pharma (Shanghai, China). Human CDK6 eukaryotic overexpression vector, mouse CDK6 shRNA Adeno-associated Viral virus (AAV), and mouse CDK6 overexpression AAV all were purchased from Gene Pharma (Shanghai, China). Overexpression hsa_circ_0002062 and shRNA hsa_circ_0002062 plasmids were purchased from Gisai Biotechnology Co., Ltd. (Guangzhou, China). The FuGENE HD Transfection Reagent (Promega Corp., United States) was used in PASMCs with specific shRNAs and plasmid vectors based on the provided instructions. The sequences of the shRNA primers are as follows: Sh-CDK6: 5′-GUUUGAACAUGUCGAUCAATT-3′; sh-NC: 5′-UUCUCCGAACGUGUCACGUTT-3′;

hsa-miR-942-5P inhibitor: 5′-CACAUGGCCAAAACAGAG AAGA-3′; hsa-miR-942-5p mimics: 5′-UCUUCUCUGUUUU GGCCAUGUG-3′; miR-N.C

5′-CAGUACUUUUGUGUAGUACAA-3′.

### Ethynyldeoxyuridine (Edu) Analysis

Edu incorporation assays were used to determine cell proliferative abilities. Cells in logarithmic growth were taken and cultured in 6-well plates. Edu labeling solution (20 μM, Beyotime, Shanghai, China) was added to the 6-well plates 48 h after transfection and then incubated for 2 h at 37°C and 5% CO_2_. The cells were stained with the Click Additive Solution (Beyotime, Shanghai, China) and Hoechst 33342, and an anti-Edu working solution according to the manufacturer’s instructions. After this, cells were treated with 4% paraformaldehyde and PBS containing 0.3% Triton X-100. The percentage of Edu-positive cells was finally measured using fluorescence microscopy analysis.

### Apoptosis Analysis by TUNEL

Approximately 5 × 10^7^ cells/ml were fixed in 4% paraformaldehyde for 30 min and further treated with PBS containing 0.3% Triton X-100. The samples were first incubated in a TUNEL solution at 37°C for 1 h and stained according to the manufacturer’s instructions (Roche, United States). TUNEL-positive myocytes were determined using the IMS Image Analysis System (Kilton Biotechnology, China), and the apoptotic rate was then calculated based on the number of the TUNEL-positive cells.

### Transwell Migration Assay

Cell migration was determined using a transwell chamber (Corning Costar, United States). Cells were first cultivated in a 24-well transwell plate in starvation medium for 24 h. For migration assays, 5 × 10^4^ cells/well were seeded into the top chamber containing 0.2-ml serum-free DMEM. After incubation for 24 h, the cells migrated to the bottom chamber containing 0.7-ml medium supplemented with 10% FBS. The cells that did not migrate were carefully swabbed out of the chamber. The cells were fixed with 4% paraformaldehyde solution and stained with 0.5% crystal violet, imaged, and migration rates were calculated using an Olympus microscope (Tokyo, Japan).

### Western Blotting

Cells were homogenized in an ice-cold RIPA buffer (Keelton Co., Ltd., China). Protein concentrations were determined using a bicinchoninic acid (BCA) protein assay kit (Thermo, Rockford, United States). Equal amounts of protein were separated by 10% sodium dodecyl sulfate-polyacrylamide gel electrophoresis (SDS-PAGE) (Bio-Rad, Hercules, CA, United States) and transferred onto polyvinylidene difluoride (PVDF) membranes (Merck Millipore, Darmstadt, Germany). The membranes were blocked with 5% non-fat dry milk in Tris-buffered saline with 0.05% Tween 20 (TBST) at room temperature for 2 h, incubated overnight with the primary anti-CDK6 (1:1,000 dilution, ab241554; Abcam), anti-VEGF (1:2,000, GTX102643; GeneTex), antiAT1R (1:2,000, ab124734; Abcam), or anti-GAPDH antibodies (1:2,000 dilution, #5174;CST). After washing with TBST three times for 10 min, the membranes were treated with HRP-labeled secondary antibodies (1:1,000 dilution, Beyotime, China) for 1 h and visualized using the chemiluminescence method (Thermo, Rockford, United States). The relative protein expression was measured using GAPDH as an internal control.

### RNA Binding Protein Immunoprecipitation (RIP)

RIP assays were conducted using the EZ-Magna RIP kit (Millipore, Billerica, MA, United States). hPASMCs at 80–90% confluency were lysed in a full lysis buffer and then incubated with a RIP buffer containing magnetic beads conjugated with an anti-Ago2 antibody (Abcam, United States) or a negative control IgG antibody (Millipore, United States). The samples were incubated with 150-μl Proteinase K buffer at 55°C for 30 min to remove protein and purify RNA. Next, the immunoprecipitated RNA was reversed transcribed and quantified by qRT-PCR.

### Dual-Luciferase Reporter Gene Assays

Dual-luciferase reporter assays were performed to verify further whether hsa_circ_0002062 or CDK6 was the target of hsa-miR-942-5P.

hsa_circ_0002062 wild type, hsa_circ_0002062 mutant, CDK6 wild type, or CDK6 mutant inserts were inserted into the pGL3-basic luciferase vector (Promega, Madison, WI, United States) in order to generate report plasmids. Afterward, cells transfected with indicated reporter plasmid were cultured in a 6-well plate and co-transfected with 5-μL miR-NC, or hsa-miR-942-5p mimic using Lipofectamine 2000 (Invitrogen). The Dual-Luciferase Reporter Gene Assay System (E1910, Promega, United States) was used to detect luciferase activity according to the manufacturer’s instruction, and the ratio of firefly to renilla luciferase activity was calculated.

### Establishment of an HPH Mouse Model and *in vivo* Experiments

In order to construct CDK6 overexpression vector, the coding region of CDK6 (NM_009873) gene was inserted into pAAV-CMV-MCS vector carrying CMV promoter. To build CDK6 shRNA vector, the sequence of sh-CDK6 (5′-GGATATGATGTTTCAGCTTCTCGAGAAGCTGAAACATCA TATCCTTTTT—3′) was inserted into pAAV-U6-MCS carrying the U6 promoter. The CDK6 overexpression vector and CDK6 shRNA vector were co-transfected into AAV-293 cells with a packer plasmid (pAAV-RC) and a helper plasmid (phelper), respectively. Mouse CDK6 overexpression AAV and mouse CDK6 shRNA AAV were packaged and collected, while AAV without target gene was collected as controls.

All the animal experiments were performed with the approval of the Animal Experiment Ethics Committee of Renji Hospital affiliated with Shanghai Jiao Tong University. A total of 42 healthy male C57/BL6 mice 6–9 weeks old and weighing 22–26 g were purchased from SIPPR-BK Lab Animal Co., Ltd. (Shanghai, China), with six mice in the normoxic group and six mice in the hypoxic group. Mice in the Hypoxia + Vector group, the Hypoxia + CDK6 group, and the Hypoxia + sh-CDK6 group were inoculated with control AAV (100 μl,1 × 10^11^ vg/ml),CDK6 overexpression AAV (100 μl,1 × 10^11^ vg/ml). and CDK6 shRNA AAV (100 μL, 1 × 10^11^ vg/mL), respectively. Mice in the Control group were fed under normoxia. All animals in each group were repeatedly inoculated with AAVs through tail vein once a week during modeling. Four weeks later, the mice were sacrificed, and several indices were measured. At the end of the experiment, the lung tissues were fixed with 4% paraformaldehyde and embedded in paraffin. The vascular remodeling of mice in different groups was analyzed by HE and Masson staining. RNA and protein were extracted from the pulmonary arteries in other lung tissues. The expression levels of CDK6 mRNA were determined by qPCR, while the expression levels of CDK6, VEGF, and AT1R were analyzed by Western blot.

### Statistical Analyses

Statistical analyses were conducted using SPSS 22.0 (Chicago, IL, United States) and GraphPad Prism 8 (San Diego, CA, United States). All data are presented as the mean ± standard deviation (SD). Student’s *t*-test and one-way analysis of variance (ANOVA) were used to estimate significant differences between different groups. A *p*-value < 0.05 was considered to be statistically significant.

## Results

### Hypoxia Promoted the Proliferation and Migration of hPASMCs and Upregulated the Expression of Hsa_circ_0002062 and CDK6

**FIGURE 1:**
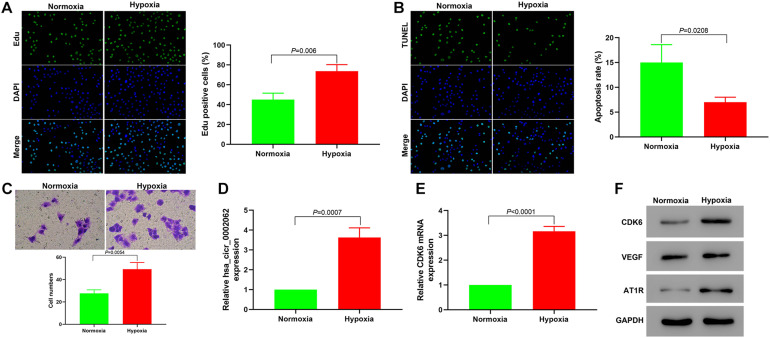
Hypoxia promotes the proliferation and migration of hPASMCs and upregulates the expression of hsa_circ_0002062 and *CDK6.* **(A–C)** Based on the results of Edu incorporation assays **(A)**, TUNEL assays **(B)**, and Transwell assays **(C)**, cell proliferation and migration were elevated, while the apoptosis rate of hypoxic PASMCs decreased under hypoxia compared to normoxic conditions (*p* < 0.01). **(D,E)** Relative expression levels of *hsa_circ_0002062* and *CDK6* mRNA were significantly upregulated in the hypoxic group compared with the control group. **(F)** Under hypoxia, the protein expression levels of CDK6, VEGF, and AT1R were significantly upregulated. Error bars represent the mean ± SD from triplicate experiments. Image magnification: ×200.

We first investigated the role of hypoxia in the proliferation and apoptosis of hPASMCs using an Edu incorporation assay and by TUNEL staining. The Edu assay revealed that hypoxia significantly increased the percentage of Edu-positive cells compared with normoxia (Figure 1A), indicating that the content of newly synthesized DNA in hPASMCs was significantly increased under hypoxia. As shown in Figure 1B, hypoxia downregulated the apoptosis ratio of cells compared with controls. This result suggested that hypoxia affected hPASMC proliferation via regulation of apoptosis. Transwell assays indicated that hypoxia significantly accelerated hPASMC migration compared with cells under normoxia (Figure 1C). These findings demonstrated that hypoxia promoted hPASMC proliferation and migration. To investigate the role of hsa_circ_0002062 in hPASMCs, we investigated the expression of hsa_circ_0002062 using qRT-PCR. The relative expression levels of hsa_circ_0002062 and CD*K6* mRNA were significantly upregulated in the hypoxic group compared with the control normoxic group (Figures 1D,E). Furthermore, the protein expression levels of CDK6, VEGF, and AT1R, which are related to vascular smooth muscle cell proliferation, were significantly elevated under hypoxia (Figure 1F).

### Downregulation of Hsa_circ_0002062 Expression in hPASMCs Under Hypoxia Attenuated Cell Proliferation and Migration

**FIGURE 2:**
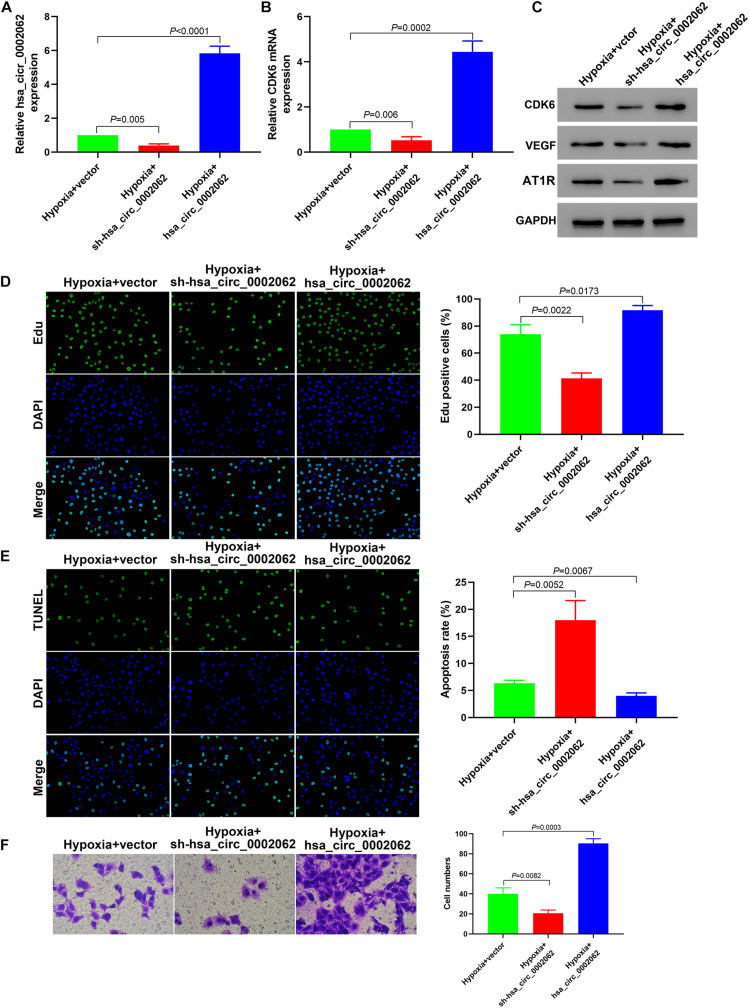
Downregulation of hsa_circ_0002062 expression in hPASMCs under hypoxia attenuates cell proliferation and migration. **(A)** qRT-PCR indicated successful silencing or overexpression of hsa_circ_0002062. **(B,C)** The mRNA levels of *CDK6* and the protein levels of CDK6, VEGF and AT1R were significantly decreased in cells treated with sh-hsa_circ_0002062, whereas they were increased in cells overexpressing hsa_circ_0002062 (all *p* < 0.01). **(D–F)** Edu assay **(D)**, TUNEL assay **(E)**, and Transwell assay **(F)**, indicating that cell proliferation and migration were elevated while the apoptosis rate in hypoxia hPASMCs was lowered in the overexpressed hsa_circ_0002062 group, in contrast to the control group. Knockdown of hsa_circ_0002062 with shRNA restrained proliferation and migration while stimulated the apoptosis of hypoxic hPASMCs. Error bars represent the mean ± SD from triplicate experiments. Image magnification: ×200.

To investigate the biological role of hsa_circ_0002062 in hPASMCs under hypoxia, we constructed plasmids containing short hairpin RNA (shRNA) targeting hsa_circ_0002062 or overexpressing hsa_circ_0002062, and then stably transfected these into hPASMCs, which we confirmed by qRT-PCR (*p* < 0.01; Figure 2A). Edu incorporation assays showed that hsa_circ_0002062 overexpression resulted in a significant increase in cell proliferation. Conversely, hsa_circ_0002062 shRNA led to a significant decrease in cell proliferation compared to that in the control group (*p* < 0.05; Figure 2D). To probe the effects of hsa_circ_0002062 overexpression or knockdown, we used TUNEL staining to investigate hPASMC apoptosis. The cell apoptosis ratio of cells overexpressing hsa_circ_0002062 was significantly decreased relative to the control group (*p* < 0.05; Figure 2E). Transwell assays revealed that migration ability was significantly increased after overexpression of hsa_circ_0002062, whereas knockdown of hsa_circ_0002062 expression via shRNA decreased the migration ability of hPASMCs (*p* < 0.05; Figure 2F). Treatment with hsa_circ_0002062 overexpression plasmid substantially increased the expression of CDK6 mRNA, a transcriptional regulator that links cell-cycle progression to differentiation and cell proliferation (Figure 2B). Western blotting analysis to measure protein expression levels indicated that knockdown of hsa_circ_0002062 led to noticeably decreased levels of CDK6, VEGF, and AT1R (Figure 2C), which are strongly associated with control of cell proliferation. The findings raise the intriguing possibility that inhibition of hsa_circ_0002062 may block the proliferation of hypoxic hPASMCs, representing a novel approach for the treatment of vascular remodeling in HPH patients.

### Hsa_circ_0002062 Acts as a Competing Endogenous RNA (CeRNA) to Upregulate the Expression of *CDK6* by Sequestering Hsa-miR-942-5p, Thereby Affecting the Biological Characteristics of Hypoxic hPASMCs

**FIGURE 3:**
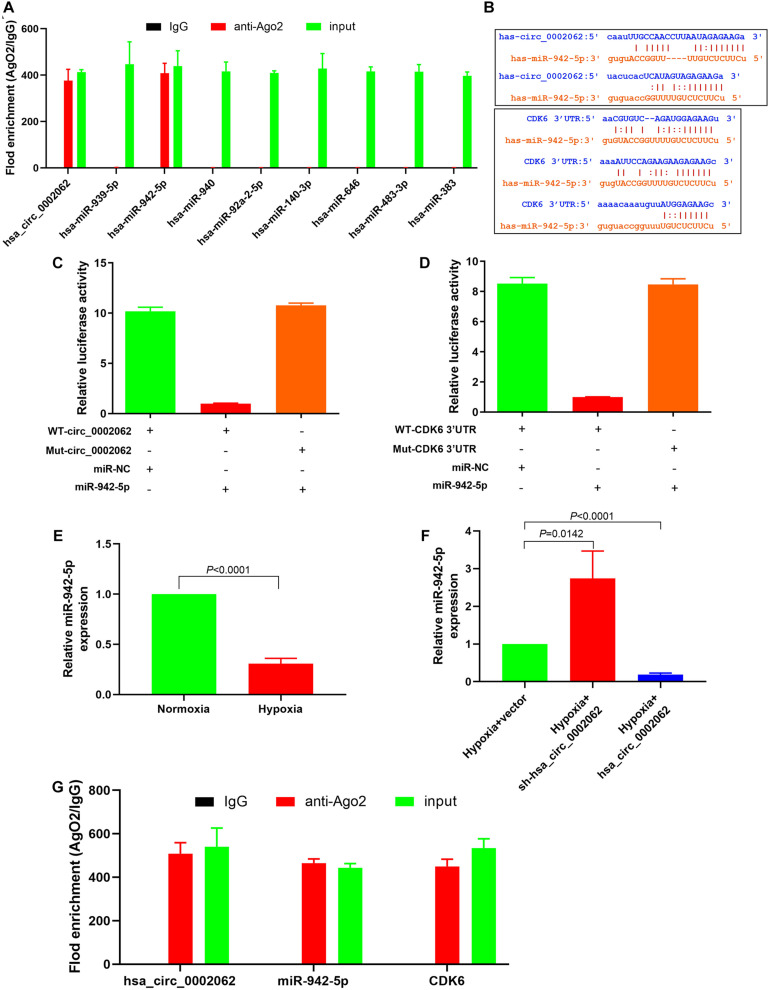
Hsa_circ_0002062 upregulates the expression of CDK6 by competitively binding to hsa-miR-942-5p. **(A)** RIP showing that hsa_circ_0002062 binds to hsa-miR-942-5p as a miRNA sponge. **(B)** ENCORI analysis indicating the binding sites common to hsa-miR-942-5p, hsa_circ_0002062 and the *CDK6* 3′UTR region. **(C,D)** Luciferase reporter assay validating the direct interaction between hsa-miR-942-5p and hsa_circ_0002062 or the CDK6 3′UTR. **(E)** The expression level of hsa-miR-942-5p in hPASMCs was significantly decreased under hypoxia. **(F)** Knockdown of hsa_circ_0002062 elevates hsa-miR-942-5p expression in hPASMCs, while overexpression of hsa_circ_0002062 inhibits the expression of hsa-miR-942-5p. **(G)** RIP showing that hsa_circ_0002062, miR-942-5p, and CDK6 were enriched in Ago2-containing precipitates. Error bars represent the mean ± SD from triplicate experiments.

Previous evidence has indicated that hsa_circ_0002062 can serve as a sponge to regulate gene expression via sequestering miRNAs in CTEPH. The Encyclopedia of RNA Interactomes (ENCORI) was used to predicate the potential miRNAs that may bind to hsa_circ_0002062. Eight miRNAs were found that be possible targets of hsa_circ_0002062. The binding of circRNAs to miRNAs is mediated by Argonaute 2 (Ago2). We therefore used RNA immunoprecipitation (RIP) with an antibody against Ago2 in PASMCs, and determined whether the 8 miRNAs related to hsa_circ_0002062 found in previous studies were enriched by RIP using qPCR. The RIP results showed that hsa_circ_0002062, has-miR-942-5p, and CDK6 were enriched in Ago2-containing immunoprecipitates (Figure 3G). Our findings indicated that Ago2 antibody could simultaneously bind hsa_circ_0002062 and hsa-miR-942-5P in hypoxic PASMCs, indicating that Ago2 mediated the interaction of hsa_circ_0002062 with hsa-miR-942-5P (Figure 3A). ENCORI analysis indicated that there were binding sites common to hsa-miR-942-5p, hsa_circ_0002062, and the CDK6 3′UTR region (Figure 3B).

Furthermore, a dual-luciferase reporter assay was performed to assess the effect of hsa_circ_0002062 on hsa-miR-942-5P activity and test whether CDK6 was the target of hsa-miR-942-5P. The luciferase assay results showed that the luciferase activity was reduced in hPASMCs co-transfected with miR-942-5P and WT-hsa_circ_0002062 or WT-CDK6 3′ UTR but was not reduced in hPASMCs containing MUT-hsa_circ_0002062 (Figure 3C) or MUT-CDK6 3′UTR (Figure 3D). These results confirmed that hsa_circ_0002062 directly sequestered hsa-miR-942-5p, and that hsa-miR-942-5p targets CDK*6*. As illustrated in Figure 3E, the expression level of hsa-miR-942-5p in hPASMCs was significantly decreased under hypoxia. To further corroborate the effect of hsa_circ_0002062 on hsa-miR-942-5P activity, qRT-PCR analysis showed that knockdown of hsa_circ_0002062 triggered a significant increase in hsa-miR-942-5p expression in hypoxic hPASMCs, while overexpression of hsa_circ_0002062 under hypoxia inhibited the expression of hsa-miR-942-5 (Figure 3F). Altogether, we found that hsa_circ_0002062 could sequester hsa-miR-942-5p as a CeRNA and inhibit its activity, and that the hsa_circ_0002062/hsa-miR-942-5P/CDK6 signaling pathway may be involved in hypoxia responses in PASMCs.

### Hsa-miR-942-5p Reverses Hsa_circ_0002062-Mediated Promotion of Cell Proliferation and Migration

**FIGURE 4:**
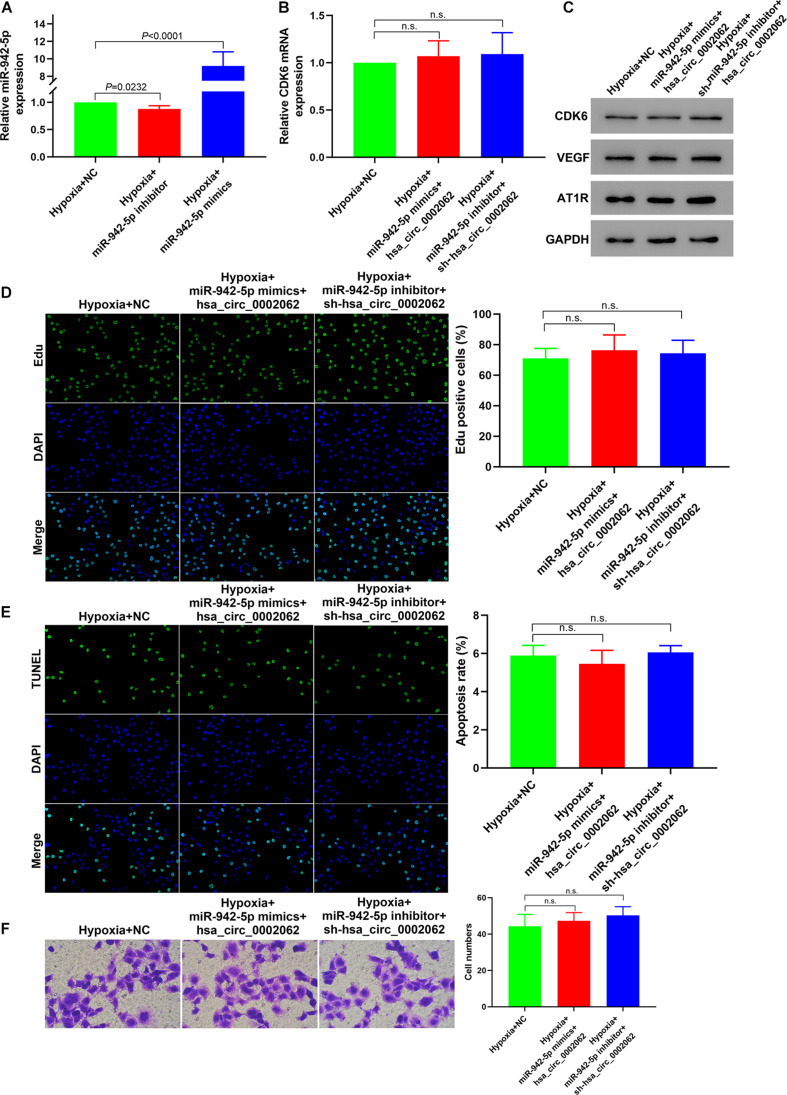
Hsa-miR-942-5p reverses the hsa_circ_0002062-mediated promotion of cell proliferation and migration. **(A)** qRT-PCR indicated successful silencing and overexpression of hsa-miR-942-5p. **(B–F)** Co-transfection of miR-127-5p inhibitor and hsa_circ_0002062 siRNA, or hsa-miR-942-5p mimic and hsa_circ_0002062-overexpression plasmid in hPASMCs under hypoxia. There were no significant changes between groups in the expression level of *CDK6* mRNA **(B)** protein levels of CDK6, VEGF and AT1R **(C)**, cell proliferation **(D)**, migration **(E)**, or cell apoptosis **(F)**. Image magnification: ×200. n.s.: no significant.

Our further study focused on the involvement of hsa-miR-942-5p with respect to the functionality of hsa_circ_0002062 in hypoxic PASMCs. Having confirmed that hsa-miR-942-5p expression was negatively regulated by hsa_circ_0002062 expression, we further investigated whether hsa-miR-942-5p reversed hsa_circ_0002062-mediated promotion of hPASMC proliferation and migration. After transfection with hsa-miR-942-5p inhibitor in hPASMCs under hypoxia, the expression level of hsa-miR-942-5p was decreased (*P* = 0.0232). Transfection of hPASMCs with hsa-miR-942-5p mimic significantly upregulated the expression level of hsa-miR-942-5p under hypoxia (*P* < 0.0001), which was confirmed by qRT-PCR (Figure 4A). As illustrated in Figure 2, knockdown of hsa_circ_0002062 with shRNA significantly restrained proliferation and migration and stimulated the apoptosis of hypoxic PASMCs, while transfection with hsa_circ_0002062-overexpression plasmid had the opposite effect. In order to investigate whether hsa_circ_0002062 exerted its oncogene effect through sequestration activity on hsa-miR-942-5p, we co-transfected hPASMCs with an hsa-miR-942-5p inhibitor and hsa_circ_0002062 shRNA (Figure 4B). The effects of hsa_circ_0002062 knockdown on the proliferation, migration, and apoptosis of hPASMCs were reversed by the hsa-miR-942-5p inhibitor (Figures 4D–F). Importantly, the inhibition of the protein expression of CDK6, VEGF, and AT1R by hsa_circ_0002062 shRNA was attenuated by the hsa-miR-942-5p inhibitor (Figure 4C). In addition, we co-transfected hPASMCs with hsa-miR-942-5p mimic and hsa_circ_0002062-overexpression plasmid (Figure 4B). The results were consistent with the above findings. The effects of overexpression of hsa_circ_0002062 on the proliferation, migration, and apoptosis of hPASMCs, as well as the protein levels of CDK6, VEGF, and AT1R, were all rescued by hsa-miR-942-5p mimic (Figures 4B–F). Taken together, these results clarified that hsa-miR-942-5p was negatively correlated with hsa_circ_0002062 expression and reversed the hsa_circ_0002062-mediated promotion of cell proliferation or migration and inhibition of cell apoptosis.

### *In vitro* Downregulation of CDK6 Reverses Hypoxia-Mediated Promotion of Cell Proliferation and Migration

**FIGURE 5:**
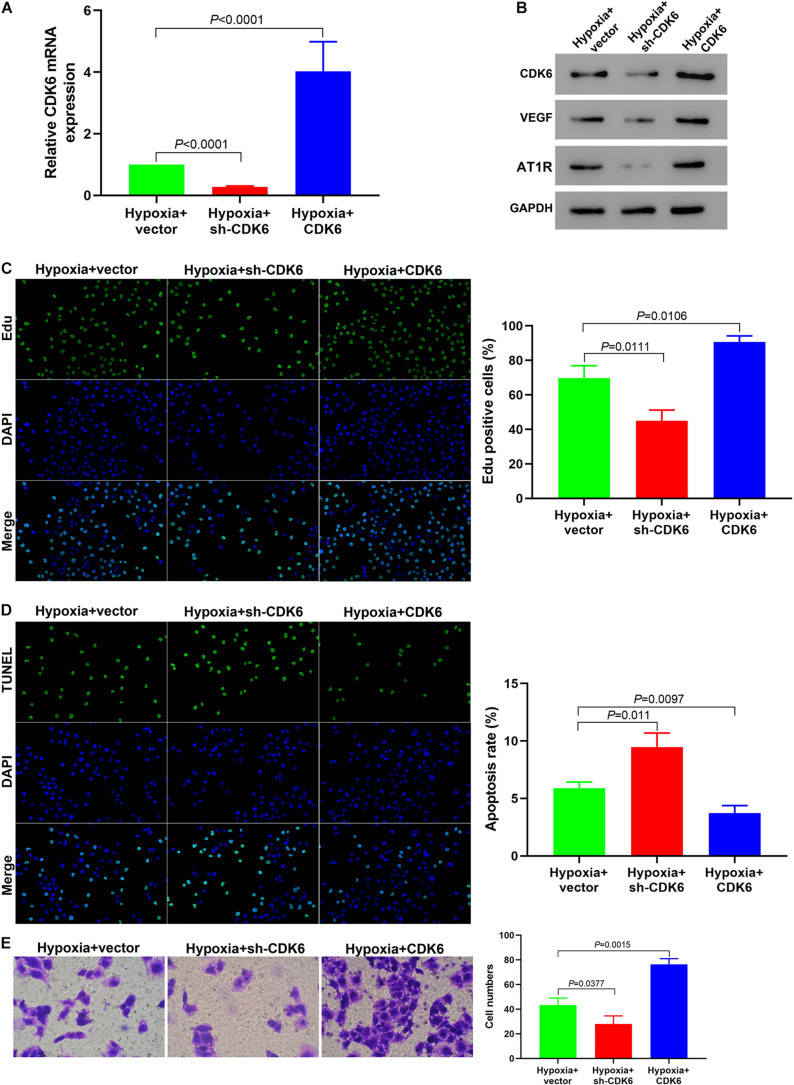
Downregulation of CDK6 *in vitro* reverses the hypoxia-mediated promotion of cell proliferation and migration. **(A)** qRT-PCR indicating successful silencing and overexpression of *CDK6*. **(B)** The protein expression of VEGF and AT1R was significantly decreased in hPASMCs treated with sh-CDK6. In contrast, they were elevated in cells treated with CDK6 overexpression vectors (all *p* < 0.01). **(C–E)** Edu assay **(C)**, TUNEL assay **(D)**, and Transwell assay **(E)** indicating that the cell proliferation and migration were decreased while the apoptosis rate of hypoxic PASMCs was elevated in the sh-CDK6 group under hypoxia, compared to the control group. Error bars represent the mean ± SD from triplicate experiments. Image magnification: ×200.

Our results from qRT-PCR confirmed the successful silencing and overexpressing of CDK6 under hypoxia (Figure 5A). The downregulation of CDK6 decreased the expression levels of VEGF and AT1R in hypoxic hPASMCs, while the upregulation of CDK6 had the opposite effect (Figure 5B). The proliferation of hPASMCs in the sh-CDK6 group significantly decreased compared to that in the CDK6 overexpression group (Figure 5C). Moreover, hPASMCs treated with sh-CDK6 exhibited a higher ratio of apoptosis than cells treated with overexpressing plasmid targeting CDK6 (Figure 5D). Further, knockdown of CDK6 with sh-RNA led to decreased cell migration compared with the overexpressed CDK6 group (Figure 5E). These results clarified that CDK6 promoted hPASMC proliferation and migration and knockdown of CDK6 reversed this hypoxia-mediated cell proliferation and migration.

### Downregulation of CDK6 *in vivo* Retarded Pulmonary Vascular Remodeling in a Murine Model of HPH

**FIGURE 6:**
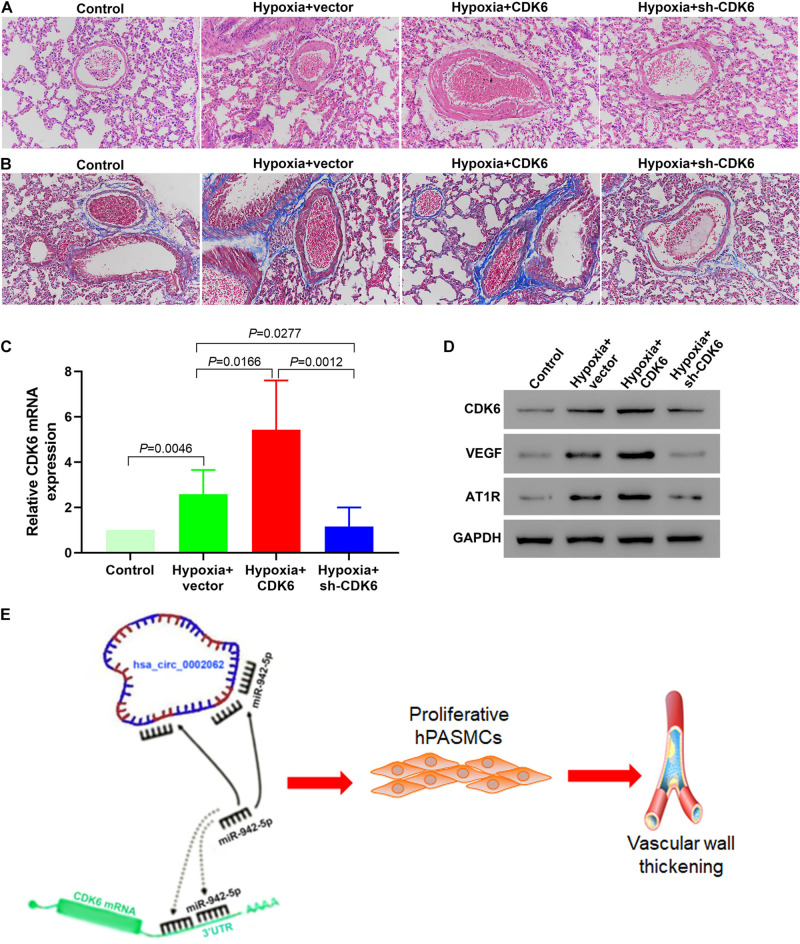
Low Expression of CDK6 in mice retarded pulmonary vascular remodeling in the development of murine HPH. **(A)** HE staining demonstrating that knockdown of *CDK6* with shRNA decreased the thickness of the pulmonary artery wall in mice. In contrast, transfection with a CDK6-overexpression plasmid had the opposite effect. **(B)** Masson’s trichrome staining revealing that upregulated CDK6 expression increased collagen fiber precipitation in the pulmonary artery wall of mice, while down-regulation of CDK6 expression did not. **(C)** The expression of *CDK6* mRNA in the pulmonary arteries of mice in different groups by qRT-PCR. **(D)** Protein expression levels of CDK6, VEGF and AT1R in the pulmonary arteries of mice in each group by Western blot. **(E)** As illustrated in the schematic diagram, hsa_circ_0002062 functions as an endogenous hsa-miR-942-5P sponge to sequester and inhibit hsa-miR-942-5P activity and increase CDK6 expression, which resulted in hPASMCs proliferation and pulmonary vascular wall thickening. Error bars represent the mean ± SD from triplicate experiments. Image magnification: ×200.

We investigated the *in vivo* activity of CDK6 in a mice HPH model. To confirm whether CDK6 affects pulmonary vascular remodeling in murine development of HPH, we examined hPASMCs transfected with shRNA and overexpressing plasmid targeting CDK6. HE staining results demonstrated that transfection with this CDK6-overexpression plasmid increased the thickness of the pulmonary artery wall in mice. In contrast, the thickness was significantly decreased in the sh-CDK6 group (Figure 6A). Furthermore, Masson’s trichrome staining findings confirmed the results from HE staining and revealed that overexpression of CDK6 increased collagen fiber precipitation in the murine pulmonary artery wall, while knockdown of CDK6 had the opposite effect (Figure 6B). To test this hypothesis, we performed real-time qRT-PCR and Western blotting to evaluate the transcription and protein expression levels of CDK6 after transfection. Our data showed that the CDK6 in mRNA and protein levels decreased compared with control groups after transfection of CDK6-shRNA, whereas the CDK6 expression levels significantly increased upon overexpression of CDK6 (*p* < 0.05; Figures 6C,D). Overexpression of CDK6 also increased the protein expression levels of VEGF and AT1R (Figure 5C). These results suggested that VEGF and AT1R were upregulated by CDK6 at the translational level. Our findings suggested that CDK6 was involved in developing pulmonary vascular wall remodeling in HPH and promoted hPASMCs proliferation *in vivo*. Taken together, as illustrated in the schematic diagram, hsa_circ_0002062 functioned as an endogenous hsa-miR-942-5P sponge to increase CDK6 expression, which resulted in hPASMCs proliferation and pulmonary vascular wall remodeling (Figure 6E).

## Discussion

An accumulated number of recent studies have identified that circRNAs are intensively associated with various respiratory diseases such as lung cancer, acute respiratory distress syndrome, pulmonary tuberculosis, pulmonary hypertension (PH), and silicosis (Li et al., 2020; Lu et al., 2020; Wang L. et al., 2020; Zou et al., 2020). However, these studies focused exclusively on the functions of circRNAs in lung cancers and only a few have described their functions in other respiratory diseases. New study is required to provide a better understanding of the role of circRNAs in PH.

In our study, hsa_circ_0002062 was found to be highly expressed in hypoxic PASMCs, and promoted pulmonary vascular remodeling, demonstrating that hsa_circ_0002062 might promote HPH development. *In vitro* experiments demonstrated that when hsa_circ_0002062 was upregulated, cell proliferation was induced, and cell apoptosis was inhibited. Meanwhile, when hsa_circ_0002062 was downregulated, the results were opposite. As hsa_circ_0002062 may play a stimulative role in HPH, these results supported the hypothesis that downregulating hsa_circ_0002062 inhibits HPH progression.

CircRNAs, as a recent identified member of the non-coding RNA family, will be a promising diagnostic and therapeutic target in PH (Gokool et al., 2020). A series of cirRNAs have been found to participate in the development of PH, but only a few of them are clearly understood in terms of their distinct pathophysiological mechanisms (Zhang et al., 2020). In order to better understand the in-depth mechanisms behind the pathogenesis of PH, we designed this study with the aim of investigating the roles of hsa_circ_0002062 in the viability and migration of hypoxic PASMCs via hsa-miR-942-5P-mediated regulation of CDK6. Collectively, we revealed that hsa_circ_0002062 upregulated the expression of CDK6 by binding to hsa-miR-942-5P, consequently affecting the cellular pathophysiological processes of hypoxic PASMCs, such as apoptosis, proliferation, and migration. Our results provide new insights into hsa_circ_0002062 in the pathogenesis of HPH. Downregulation of hsa_circ_0002062 has the potential to block pulmonary vascular remodeling induced by CDK6, overexpression of which accelerates cell proliferation in pulmonary hypertension.

Studies on circRNAs have opened a new chapter in respiratory diseases, as they act as endogenous miRNAs sponges to bind miRNAs through their binding sites to prevent their interactions with target genes (Wang et al., 2019). Liu et al. (2018) demonstrated a novel regulatory signaling pathway involving hsa_circRNA_103809/miR-4302/ZNF121/MYC in lung cancer progression. Additionally, an increasing number of studies have flagged the potential axis of circRNA-miRNA-mRNA as an emerging regulator of non-cancer diseases (Hall et al., 2019; Ma et al., 2020; Miao et al., 2020; Xue et al., 2020). Hall et al. (2019) have suggested that circ_Lrp6 enriched in Vascular Smooth Muscle Cells was a modulator and natural sponge of miR-145. Miao et al. (2020) found that hsa_circ_0046159 serves as a crucial regulator of the development of *CTEPH* within the miRNA-circRNA network. Nevertheless, previous studies are predominantly phenomenological, in that they are mostly profiling dysregulated circRNAs and lack in-depth mechanisms. Our result revealed that hsa_circ_0002062 acts as hsa-miR-942-5P sponge to upregulate *CDK6* in HPH. Further experiments showed that knockdown of hsa_circ_0002062 attenuated pulmonary vascular remodeling, inhibited cell growth, and promoted cell apoptosis.

It has been revealed that miR-942-5p plays an important role in many cell types (Zhang et al., 2017; Luo et al., 2020; Rao et al., 2020). miR-942-5p regulates septic acute kidney injury by targeting FOXO3 (Luo et al., 2020). Five upregulated miRNAs, including miR-942-5p, were identified by qRT-PCR in breast cancer patients’ based on microRNA profiling from blood samples (Zhang et al., 2017). It should be noted that miR-942-5p has been shown to play an increasingly vital role in a series of respiratory diseases (Scheicher et al., 2015; Yang et al., 2019; Wang Q. et al., 2020). It has been reported that miR-942-5p promotes tumor migration and invasion via ZNF471 in non-small cell lung cancer (NSCLC) (Wang Q. et al., 2020). Yang et al. (2019) demonstrated the tumor-promoting activity of miR-942 in NSCLC. Additionally, 761 (e.g., hsa-miR-942-5p) and 453 (e.g., hsa-miR-940) miRNAs were shown to be regulated by hsa_circ_0002062 and hsa_circ_0022342 in the CTEPH group, respectively (Miao et al., 2017). This study indicated that the hsa_circ_0002062–hsa-miR-942-5P–CDK6 and hsa_circ_0022342–hsa-miR-940–CRKL–ErbB signaling pathways might be key mechanisms in CTEPH development. The above scientific findings have highlighted the potential of the circRNA-miRNA-mRNA axis as a promising regulatory mechanism in respiratory diseases. Our study unveiled that miR-942-5p targets CDK6 in hypoxic pulmonary hypertension, which has been found to regulate the cell cycle and promote cell proliferation. Previous publications and our current studies strongly indicate that miR-942-5p is an important miRNA that is involved in various physiological and pathological processes. Thus, it becomes necessary to unearth the downstream targets of miR-942-5P in detail, which should provide further insight into the regulative mechanism of the miR-942-5P/CDK6 axis in HPH.

CDK6 is a member of the cyclin-dependent kinase (CDK) family, which governs cell cycle transitions during quiescence, senescence, and differentiation (Scheicher et al., 2015). CDK6 can promote cancer cell proliferation in different types of cancers (Nebenfuehr et al., 2020). Recent studies have shed new light on several unexpected roles for CDK6 and suggest a novel function for CDK6 as a versatile player in transcriptional regulation and differentiation, the integration of signaling networks, and gene expression modulation (Tigan et al., 2016). It has also been revealed that a number of miRNAs target CDK6 and control cell proliferation and differentiation (Zhu et al., 2016). The current study found that CDK6 was negatively regulated by hsa-miR-942-5P and positively by hsa_circ_0002062 via a specific target site within its 3′ UTR. ENCORI analysis demonstrated common binding sites in hsa-miR-942-5p, hsa_circ_0002062, and the CDK6 3′UTR region. These findings showed that CDK6 was a potential direct target of hsa-miR-942-5P and downregulating hsa-miR-942-5P led to up-regulation of CDK6.

Taken together, we found that hsa_circ_0002062 was a significant regulator of HPH development through its regulation of the hsa-miR-942-5P/CDK6 axis. Our results suggested that hsa_circ_0002062 plays a stimulative role in pulmonary vascular remodeling. It can induce PASMC proliferation by positively regulating CDK6 expression. The hsa_circ_0002062/hsa-miR-942-5P/CDK6 axis thus becomes vital to our understanding of the molecular mechanisms involved in pulmonary vascular remodeling, and may serve as a novel therapeutic target for HPH. Due to its importance, in the future it will be necessary to excavate the downstream targets of hsa_circ_0002062 in detail and gain further insight into the hsa_circ_0002062 regulation mechanism of PH.

## Data Availability Statement

The raw data supporting the conclusions of this article will be made available by the authors, without undue reservation.

## Ethics Statement

All animal experiments were performed with the approval of the Animal Care and Use Committee of Renji Hospital Affiliated to Shanghai Jiao Tong University.

## Author Contributions

YWa designed the research, wrote the manuscript, and submitted the article for publication. FH, LZ, and YL collected and analyzed the data. FH, XT, and YWu performed cell experiments. FH, YWa, and SC conducted animal experiments. All authors contributed to the manuscript and approved the submitted version.

## Conflict of Interest

The authors declare that the research was conducted in the absence of any commercial or financial relationships that could be construed as a potential conflict of interest.
